# Impact of smoking on coronavirus disease 19 severity

**DOI:** 10.1002/jmv.26456

**Published:** 2020-09-29

**Authors:** Rohin K. Reddy, Walton N. Charles, Alexandros Sklavounos, Paul T. Seed, Ankur Khajuria

**Affiliations:** ^1^ Department of Surgery and Cancer Imperial College London London UK; ^2^ Department of Women and Children's Health King's College London London UK; ^3^ Kellogg College University of Oxford Oxford UK

We thank the author for his interest^1^ in our recent systematic review and meta‐analysis^2^ and discussion of several salient points regarding research during the coronavirus disease 2019 (COVID‐19) pandemic. We agree that unprecedented volumes of new literature have unfortunately caused reductions in quality and reporting. Conclusions based on flawed analyses have been widely disseminated in both academic literature and lay media, resulting in wasted resources and potential patient harm.^3^ Indeed, in the earliest meta‐analysis assessing the effect of smoking on COVID‐19 severity,^4^ the controversial headline, “Active smoking is not associated with severity of COVID‐19,” was widely debated across scientific literature, social media, and lay press. Perhaps more insidiously, the work was also circulated by researchers and subjects financially supported by the tobacco industry.^5^ This preliminary meta‐analysis^4^ has since been found to be inaccurate, both in terms of data acquisition and statistical analysis.^6^, ^7^ Therefore, with early reservations, we prospectively registered a study protocol^8^ for a rigorously conducted, transparently reported study concerning the impact of smoking on COVID‐19 severity. Consequently, throughout conducting our review, we were fully PRISMA‐compliant,^9^ and ensured it was of high‐quality, as measured by the AMSTAR 2 criteria.^10^

Collider bias is undoubtably rife within COVID‐19 research.^11^ The author argues that the external validity of our study is undermined by selection bias, a form of collider bias, due to exclusion of non‐hospitalized patients. However, restricting analyses to solely hospitalized patients was due to the objective clinical stratification criteria for severity we used being predicated on parameters often only measured in secondary care, such as radiologic assessment and PaO_2_/FiO_2_ ratio, amongst others. Other objective measures we planned to investigate were in‐hospital outcomes, such as intensive care unit admission, mechanical ventilation requirement, and mortality. It was unlikely that detailed parameters allowing assessment of severity and in‐hospital outcomes would be available in community‐based studies. Analyzing hospitalized patients was therefore a conscious choice predefined in our a priori protocol.^8^ Therefore while not generalizable to the entire population, our review is certainly applicable to hospitalized patients with COVID‐19, which adds value when considering that approximately 20% of patients with COVID‐19 have severe or critical disease and the case fatality rate for critically ill patients is 49%.^12^ Thus, our work may allow early identification of smokers as a patient population vulnerable to worse in‐hospital outcomes, allowing timely triage and initiation of supportive measures. Nevertheless, future studies characterising the role of smoking in susceptibility to initial COVID‐19 infection, and outcomes in community settings are warranted, such that efforts to protect smokers may be co‐ordinated across the full spectrum of clinical care.

Interestingly, the author proposes that because prevalence of Chinese smokers in our study is lower than Chinese population prevalence, our sample is not representative of population smoking habits. However, caution is advised when applying this logic, as most included studies did not adjust the effect of smoking for baseline covariates and therefore comparing prevalence of smoking in hospitalized COVID‐19 patients with overall population estimates is inappropriate, as the populations may be inherently different with regard to demographic factors.^2^ Furthermore, this comparison is susceptible to collider bias, as due to sampling dependent on hospitalization, anything that influences hospitalization (ie, smoking^13^), will become negatively associated with COVID‐19 infection, thus appearing protective.^11^ This may account for “protective” effects of nicotine, alluded to by the author^1^ and reported across pre‐prints and lay media.^14^ Potential reasons for the lower reported prevalence of smoking in our study were outlined, namely misclassification bias, reverse causality, and survivorship bias.^2^

We wholeheartedly agree with the eloquent description of pitfalls involved with inferring causation from poor‐quality observational research.^1^ However, in our meta‐analysis, the majority of studies (60%) were good‐ or fair‐quality. Additionally, while the author highlights that following sensitivity analyses including only good‐quality studies, the effect of current smoking became nonsignificant, we must reiterate that there were only two studies for each outcome evaluated (severe COVID‐19 and severe or critical COVID‐19), neither of which graded severity by COVID‐19‐specific criteria, precluding meaningful interpretation. It is also likely that these particular analyses were affected by the aforementioned biases towards null results and an increased good‐quality sample size for these outcomes would be informative. The effect of a smoking history remained significant for severe disease, severe or critical disease, disease progression and mortality, even when restricting the analyses to good‐quality studies only.

Gold‐standard assessment of causality has traditionally arisen from randomized‐controlled trials (RCTs), though clearly with smoking and COVID‐19 this is an unethical option with little equipoise. Fortunately, properly performed observational studies assessing the impact of smoking have previously provided important answers.^15^ In the absence of RCT‐level evidence and issues with traditional multivariable regression,^1^ we concur that differing methodological approaches permit more precise delineation of causal relationships. We suggest that alongside triangulation, propensity score matching^16^ is used across observational research to better control for confounding, colliders and more. Future data analyzed in this way should originate from high‐quality routine collection, or bespoke prospective registries. Prospective studies established before the occurrence of outcomes of interest would minimize collider bias. As previously discussed,^2^ another option is individual‐patient‐data meta‐analysis restricted to good‐quality studies which would permit more detailed analyses and adjustment. COVID‐19 research is evolving and we hope future works will explore these new chapters, rather than attempting to close the book.

Finally, the author's concern with claiming a meta‐analysis based on poorly conducted observational data is “definitive” is valid. However, we did not state our analysis was definitive and rather, wrote that we “aimed” to definitively quantify the effects of smoking, which we considered a worthy ambition. Indeed, systematic reviews and meta‐analyses are key tools in the evidence‐based‐healthcare armamentarium, offering comprehensive summaries of the best available evidence on a given topic. Considering that almost half of all published systematic reviews now include non‐randomized studies of intervention effects,^10^ it is crucial that they are conducted and reported conscientiously, due to the aforementioned implicit biases in addition to measured and unmeasured confounders/colliders that abound in observational research. For this purpose, the AMSTAR 2 tool^10^ was specifically developed to ensure quality in studies including non‐randomized studies.

All previously published reviews investigating smoking and COVID‐19 severity range in quality from critically poor to moderate.^2^ Our meta‐analysis is the first to be deemed “high‐quality,” alongside being the largest by considerable distance. Therefore, we believe it is fair to conclude that whilst our work is by no means “definitive” on this topic, it is certainly the most definitive currently available. In the era of COVID‐19, pragmatism should reign supreme and biologically plausible effects that are clinically relevant, caused by exposures amenable to modification, must be recognized by healthcare providers, governments and policymakers to protect vulnerable patient populations and maintain public health.

## Data Availability

The data that support the findings of this study are available from the corresponding author upon reasonable request.
